# Duplex Ultrasonography for the Evaluation of Extracranial Vertebral Artery: A Prospective Comparison With Digital Subtraction Angiography

**DOI:** 10.3389/fneur.2022.814972

**Published:** 2022-06-27

**Authors:** Jie Zhang, Yingqi Xing, Li Cui

**Affiliations:** ^1^Department of Neurology, Neuroscience Center, First Hospital of Jilin University, Jilin University, Changchun, China; ^2^Department of Vascular Ultrasonography, Center of Vascular Ultrasonography, Xuanwu Hospital, Capital Medical University, Beijing, China

**Keywords:** vertebral artery stenosis, posterior circulation stroke, cerebrovascular disease, duplex ultrasonography, digital subtraction angiography

## Abstract

**Background and Objective:**

Patients with symptomatic vertebral artery stenosis are at high risk of stroke recurrence, especially ≥70% stenosis. Revascularization may be considered for extracranial vertebral artery stenosis in patients with recurrent ischemic events despite optimal medical management. Currently, there is a lack of consensus on the ultrasonic evaluation of extracranial vertebral artery stenosis in clinical practice. This study aimed to validate the efficiency of duplex ultrasonography and assess the optimal sonographic thresholds for predicting extracranial vertebral artery stenosis.

**Methods:**

This is a prospective study of all patients with symptomatic posterior circulation stroke/transient ischemic attack who were scheduled to undergo digital subtraction angiography from April 2020 to October 2021. A total of 544 vertebral arteries with a normal lumen or extracranial stenosis confirmed with digital subtraction angiography were included in the study. The peak systolic velocity at the V1 segment (PSVv1) and the V2 segment (PSVv2) were measured and the PSVv1/PSVv2 and PSVv2/PSVv1 ratios were calculated. The cutoff values were determined using receiver operating characteristic analysis.

**Results:**

The areas under the receiver operating characteristic curve of all the velocity parameters to predict extracranial vertebral artery stenosis were >0.80. The cutoff values for predicting ≥50% and ≥70% V1 segment stenosis were PSVv1 ≥146 cm/s (sensitivity 76.2%, specificity 86.3%) and PSVv1/PSVv2 ratio ≥2.2 (sensitivity 84.3%, specificity 77.6%), and PSVv1 ≥184 cm/s (sensitivity 80.8%, specificity 87.1%) and PSVv1/PSVv2 ratio ≥3.5 (sensitivity 79.5%, specificity 90.5%), respectively. The cutoff values for predicting ≥50% and ≥70% V2 segment stenosis were PSVv2 ≥80 cm/s (sensitivity 75.0%, specificity 91.0%) and PSVv2/PSVv1 ratio ≥1.2 (sensitivity 75.0%, specificity 94.8%), and PSVv2 ≥111 cm/s (sensitivity 81.0%, specificity 95.0%) and PSVv2/PSVv1 ratio ≥1.7 (sensitivity 81.0%, specificity 96.6%), respectively.

**Conclusion:**

Symptomatic patients with the ultrasonic parameters of PSVv1 ≥146 cm/s and PSVv1/PSVv2 ratio ≥2.2 at V1 segment or PSVv2 ≥80 cm/s and PSVv2/PSVv1 ratio ≥1.2 at V2 segment need to be considered for further verification by digital subtraction angiography to seek revascularization. If the parameters increase to PSVv1 ≥184 cm/s and PSVv1/PSVv2 ratio ≥3.5 at the V1 segment or PSVv2 ≥111 cm/s and PSVv2/PSVv1 ratio ≥1.7 at the V2 segment, these patients have an increased risk of recurrent stroke and are more likely to need revascularization. The results can be used as a reference for the assessment and long-term management of patients with extracranial VA stenosis.

## Introduction

Up to 20% of ischemic cerebrovascular events involving the posterior circulation are related to vertebral artery (VA) disease (1). Data from the Oxford Vascular Study and St. George Study showed that symptomatic ≥50% VA stenosis was a strong independent predictor of stroke recurrence (2). Furthermore, stroke patients with ≥70% responsible artery stenosis are at a higher risk of stroke recurrence (3–6). Endovascular treatment in extracranial VA stenosis appears safe and beneficial, with low complication rates, when compared to intracranial VA stenosis, which is more suitable for medical therapy (7–11). In patients with symptomatic extracranial VA stenosis, revascularization may be considered for ≥50% stenosis of the lesion in patients with recurrent ischaemic events despite optimal medical management (1). The majority of asymptomatic patients do not require revascularization procedures. However, there are a few groups of asymptomatic patients that have indications for treatment because of their elevated risk of stroke. It includes patients with bilateral VA stenosis ≥70%, or with unilateral VA stenosis ≥70% in the presence of an occluded or hypoplastic contralateral VA, and patients with significant dependence on collateral flow from the posterior circulation, such as in cases of carotid occlusion (12, 13). Therefore, the accurate evaluation of extracranial VA stenosis is particularly important, including ≥50% stenosis and ≥70% stenosis.

Duplex ultrasonography (DUS) is the first-line examination for patients with cerebrovascular disease not only due to its convenience and noninvasiveness but also since it can be used for real-time evaluation at the bedside, especially for critically ill patients. In patients with known VA stenosis, it is reasonable to use DUS to assess stenosis progression and to follow patients after revascularization therapies (1). Currently, Stroke Outcomes and Neuroimaging of Intracranial Atherosclerosis (SONIA) criteria are widely used to predict the intracranial VA stenosis by transcranial Doppler, while there is a lack of consensus on the ultrasonic evaluation of extracranial VA stenosis in clinical practice (14). Only two retrospective studies evaluated ≥70% stenosis of the V1 segment of VA, and they showed conflicting results (15, 16). In addition, there is a paucity of published literature on the ultrasonic evaluation of V2 and V3 segments of VA. Thus, it is necessary to carry out a prospective study with a large sample size to completely evaluate extracranial VA stenosis by DUS.

Therefore, the purpose of this study was to prospectively evaluate extracranial VA stenosis using DUS, with digital subtraction angiography (DSA) as the reference. We aimed to validate the sensitivity and specificity of DUS and assess the optimal thresholds for ≥50% and ≥70% extracranial VA stenosis. Furthermore, the positive predictive value [PPV], negative predictive value [NPV], and accuracy were calculated to thoroughly validate DUS's efficiency.

## Materials and Methods

### Population

**Figure 1:**
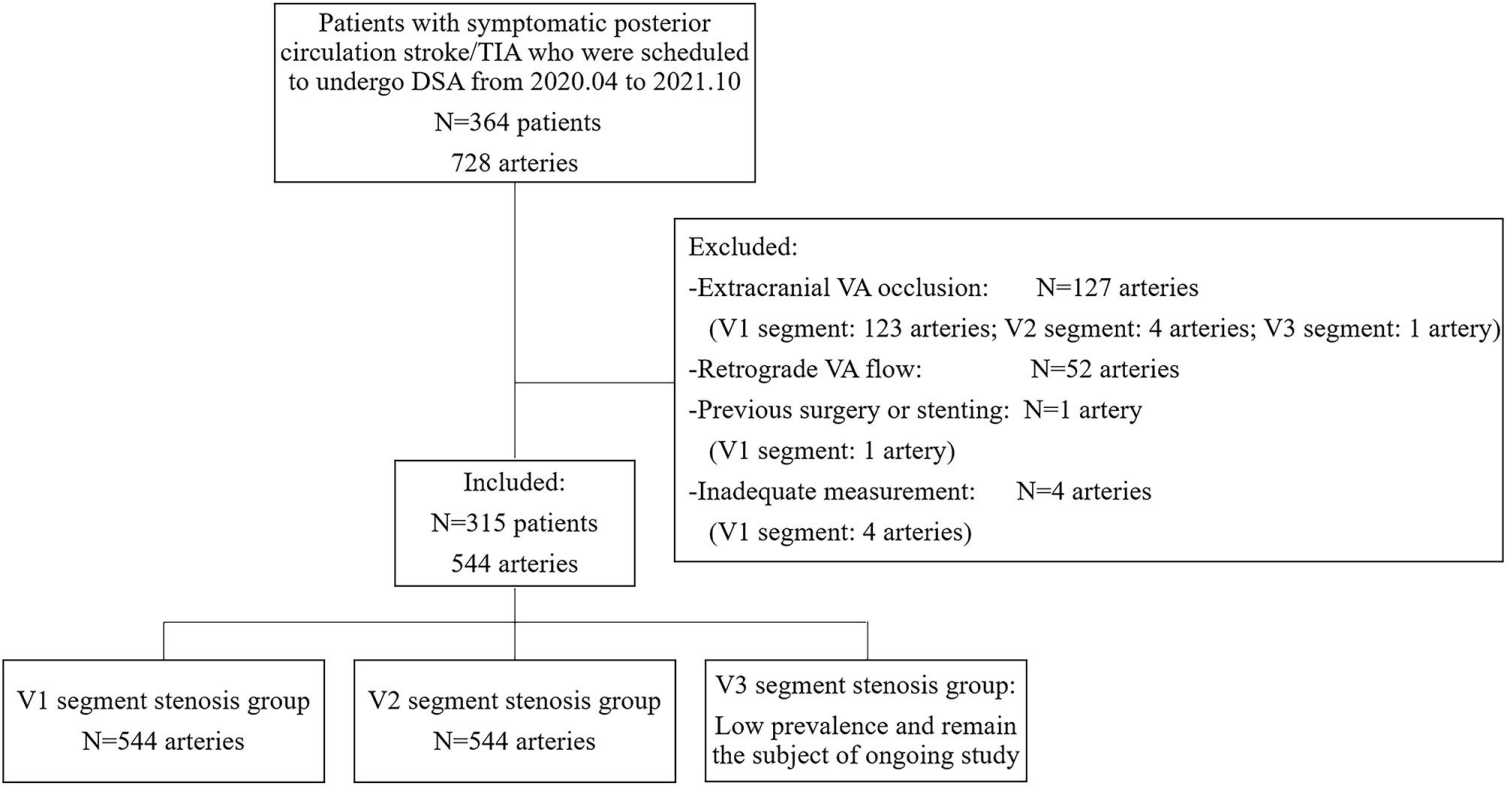
Study inclusion/exclusion criteria. TIA, transient ischemic attack; DSA, digital subtraction angiography; VA, vertebral artery.

A blind and prospective study was performed at the Stroke Unit of the Department of Neurology at the First Hospital of Jilin University from April 2020 to October 2021. Patients with symptomatic posterior circulation stroke/transient ischemic attack (TIA) who were scheduled to undergo DSA were included. All the patients had undergone computed tomographic angiography or magnetic resonance angiography examinations, which confirmed VA stenosis. The subsequent DSA was performed with a view to endovascular percutaneous transluminal angioplasty or stent implantation in the same session. Patients were recruited consecutively after informed consent, and they were evaluated by blinded examiners, first by DUS, and then by DSA, within an interval of 24 h. Data on patients' demographics and vascular risk factors were collected after DUS. For statistical analyses, the right and left sides of each patient were analyzed separately. The exclusion criteria were as follows: (i) extracranial VA occlusion; (ii) retrograde VA flow due to subclavian artery stenosis; (iii) previous extracranial VA surgery or stenting; and (iv) inadequate measurement of extracranial VA by DUS. The study protocol was approved by the ethics committee of the First Hospital of Jilin University on 20 March 2020 (approval number: 2020-632). The study was completed in compliance with the current privacy regulations. Figure 1 shows the main flowchart of this study.

### Ultrasonic Examination

According to the ultrasound protocol previously described by Pellerito, all DUS were performed by the same physician who had 9 years of vascular ultrasound experience, having performed more than 2,000 vascular cases per year, and who was unaware of clinical data (other imaging data, physical examination results, laboratory results, and patient history) (17). DUS examinations were performed using a high-end ultrasound system (iU22, Philips Healthcare, Bothell, WA) with a 9-3-MHz linear array probe and a 5-1-MHz curvilinear array probe. Patients were investigated in a supine position with the neck slightly extended. First, the common carotid artery was located in the longitudinal plane with a linear array probe. The VA was then shown between the transverse processes by the posterolateral movement of the probe. Both the V1, V2, and V3 segments were examined in B-mode, color Doppler, and pulsed-wave Doppler modes. Observe along the course of VA to the origin. If the proximal VA did not display clearly, use a linear array and curvilinear array probes together. The ultrasound parameters measured included peak systolic velocity at the V1 segment (PSVv1), V2 segment (PSVv2), and the V3 segment (PSVv3) of VA. If there was a segmental lumen narrowing and increased velocity, the parameters of the stenotic segment and non-stenotic segment would all be measured. For the V1 segment stenosis group, the PSVv1/PSVv2 (PSVr1) ratio was calculated, and PSVv2 was measured at the non-stenotic segment. For the V2 segment stenosis group, the PSVv2/PSVv1 (PSVr2) ratio was calculated, and PSVv1 was measured at the non-stenotic segment. For the V3 segment stenosis group, the PSVv3/PSVv2 (PSVr3) ratio was calculated, and PSVv2 was measured at the non-stenotic segment.

### DSA Examination

Digital subtraction angiography was performed using AXIOM Artis dBa (Siemens, Erlangen, Germany). All catheterizations were performed using a transfemoral approach with standard diagnostic catheters. After aortic arch injection, selective supra-aortic (carotid and subclavian) artery injections were performed. Angiography was interpreted by two experienced neurointerventionalists, who had at least 5 years of experience in endovascular interventional therapy. If their results were inconsistent, the final decision was made by another senior neurointerventionalist, who had 12 years of endovascular interventional therapy experience. All the neurointerventionalists were blinded to the ultrasound examination findings. The information about VA was recorded, including the location and degree of stenosis or occlusion, retrograde flow, diameter, and previous stenting. VA stenosis was defined using the North American Symptomatic Carotid Endarterectomy Trial criteria, comparing the narrowest point of stenosis to the distal normal-appearing segment of the artery (18). The degree of extracranial VA stenosis was subdivided into three categories, namely, normal, ≥50%, and ≥70%. VA hypoplasia was defined as VA diameter ≤2.5 mm in the V1 and V2 segments and slimness or absence of the whole VA, or an asymmetry ratio ≥1:1.7 (19).

### Statistical Analysis

PASS (version 15.0, NCSS, East Kaysville, UT) was used for sample size estimation. Based on the assumption that the sensitivity and specificity of DUS in predicting extracranial VA stenosis were both 70.0% (15, 16, 20–25), the required sample size was calculated as 341 VAs, with an α error of 0.05 and a CI width of 10%. SPSS (version 26.0 for Windows, IBM Corp, Armonk, NY) and MedCalc (version 19.5.6 for Windows, Mariakerke, Belgium) were used for the statistical analyses. The normality of distribution was assessed using the Kolmogorov–Smirnov test. Continuous variables were expressed as mean ± SD or median (interquartile range), and categorical variables were expressed as frequencies and percentages. Categorical variables were tested using the χ^2^ tests. The optimal thresholds of various parameters for predicting extracranial VA stenosis were determined based on the maximum Youden's index by receiver operating characteristic (ROC) curve analysis. Statistical comparisons of the areas under the ROC curve (AUC) of different hemodynamic parameters were performed according to Delong's test (26). The predictive values (sensitivity, specificity, PPV, NPV, and accuracy) were calculated. Statistical significance was set at *p* < 0.05.

## Results

### Demographic Characteristics

A total of 364 patients with 728 VAs were recruited consecutively. No DSA-related complications occurred. Among the 364 patients, VA hypoplasia was detected in 183 (50.3%) patients, including 105 (28.8%) on the right side, 70 (19.2%) on the left, and 8 (2.2%) bilaterally. The prevalence of right VA hypoplasia was significantly higher than that of left VA hypoplasia (*p* = 0.002). With the combined use of linear and curvilinear array probes, almost all the extracranial VAs (724, 99.5%) were visualized. Both the remaining four cases were of V1 segments of left VAs with relatively deep anatomical positions, and the patients had relatively short necks with large neck circumference, which made it difficult to perform the DUS examination. According to DSA results and exclusion criteria, a total of 544 arteries were finally included. The median time interval between DUS examination and DSA was 19.0 h (interquartile range 5.0). On DSA, stenosis was seen in 223 V1 segments and 44 V2 segments of VAs. Since no cases of V3 segment stenosis were found, the velocity study of V3 segment stenosis was not included in this analysis. Demographic characteristics are summarized in Table 1.

**Table 1:** Demographic characteristics.

**Variable**	**Entire cohort (*N* = 544)**
Age, years, mean (SD)	63 ± 12
Men, N (frequency, %)	434 (79.8)
Comorbidity, N (frequency, %)	
Hypertension	394 (72.4)
Diabetes mellitus	283 (52.0)
Hyperlipemia	243 (44.7)
Coronary artery disease	94 (17.3)
Smoking	423 (77.8)

*Hypertension, blood pressure ≥ 140/90 mmHg; diabetes mellitus, fasting plasma glucose ≥ 7 mmol/L or 2 h postprandial plasma glucose ≥ 11 mmol/L; dyslipidemia, total cholesterol ≥ 6.22 mmol/L or triglycerides ≥ 2.26 mmol/L or low-density lipoprotein ≥ 4.14 mmol/L; smoking, smoked for >6 months*.

### V1 Segment Stenosis

**Figure 2:**
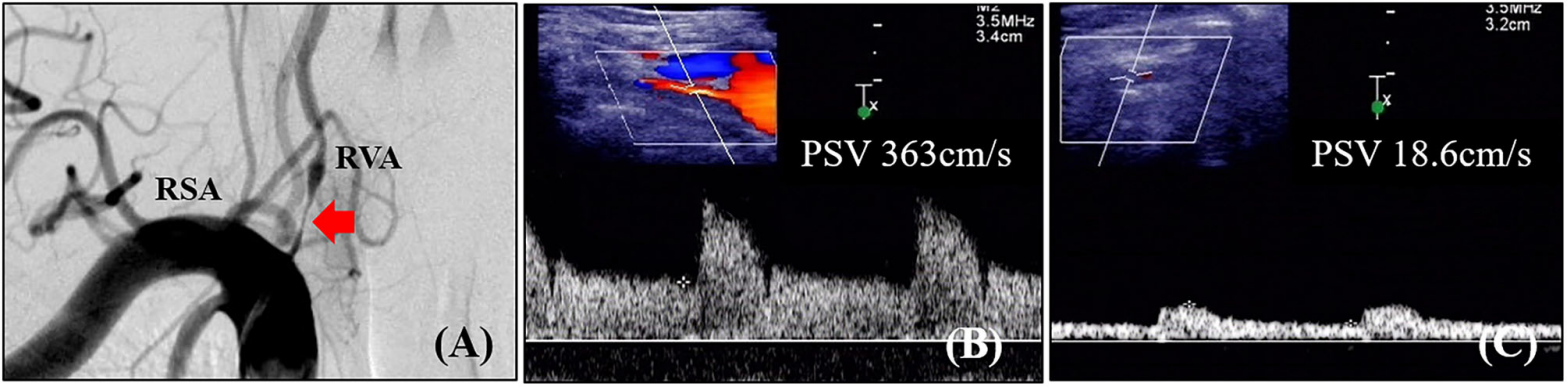
Angiographic and ultrasonic features of extracranial vertebral artery stenosis. **(A)** Digital subtraction angiography shows severe lumen narrowing of the V1 segment of the vertebral artery; **(B)** Duplex ultrasonography shows high-velocity flow at the V1 segment; **(C)** Duplex ultrasonography shows tardus flow in the V2 segment. RVA, right vertebral artery; RSA, right subclavian artery; PSV, peak systolic velocity.

**Figure 3:**
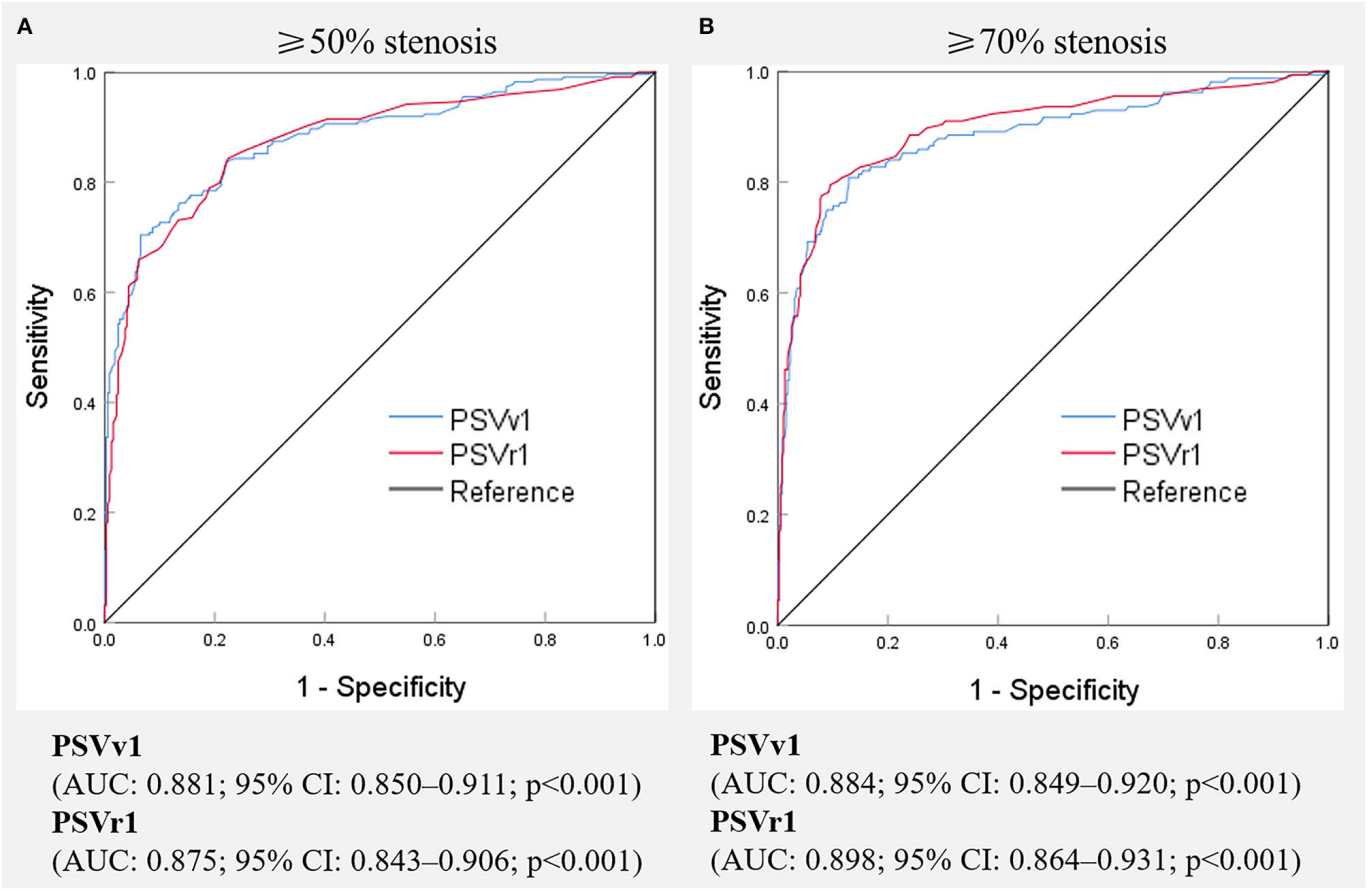
Receiver operating characteristic curves of various hemodynamic parameters for predicting ≥50% **(A)** and ≥70% **(B)** stenosis of the V1 segment of the vertebral artery. PSVv1, peak systolic velocity at V1 segment of vertebral artery; PSVr1, the ratio of peak systolic velocity at V1 segment to that at V2 segment of vertebral artery; AUC, the area under receiver operating characteristic curve, CI, confidence interval.

Digital subtraction angiography measurement of the narrowed lumen of the V1 segment of VA showed that 223 arteries (41.0%) had ≥50% stenosis, 156 arteries (28.7%) had ≥70% stenosis (Figure 2), and 321 arteries (59.0%) had normal lumen. The ROC curves and AUCs of various hemodynamic parameters for evaluating ≥50% and ≥70% stenosis of the V1 segment of VA are shown in Figure 3; all AUCs were >0.80. For evaluating ≥50% stenosis, there was no significant difference between the AUCs of PSVv1 and PSVr1 (*P* = 0.6262). The cutoff values were PSVv1 ≥146 cm/s and PSVr1 ≥2.2. For evaluating ≥70% stenosis, there was no significant difference between the AUCs of PSVv1 and PSVr1 (*P* = 0.2845). The cutoff values were PSVv1 ≥184 cm/s and PSVr1 ≥3.5. The predictive values of cutoff values are shown in Table 2.

**Table 2:** Optimal cutoff values for the evaluation of the V1 segment of the vertebral artery.

	**Sensitivity (%)**	**Specificity (%)**	**PPV** **(%)**	**NPV (%)**	**Accuracy (%)**
≥50% stenosis
PSVv1 ≥ 146 cm/s	76.2	86.3	76.9	83.6	80.9
PSVr1 ≥ 2.2	84.3	77.6	72.3	87.7	80.3
≥70% stenosis
PSVv1 ≥ 184 cm/s	80.8	87.1	71.6	91.8	85.3
PSVr1 ≥ 3.5	79.5	90.5	77.0	91.6	87.3

*PPV, positive predictive value; NPV, negative predictive value; PSVv1, peak systolic velocity at V1 segment of vertebral artery; PSVr1, the ratio of peak systolic velocity at V1 segment to that of the V2 segment of the vertebral artery*.

### V2 Segment Stenosis

**Figure 4:**
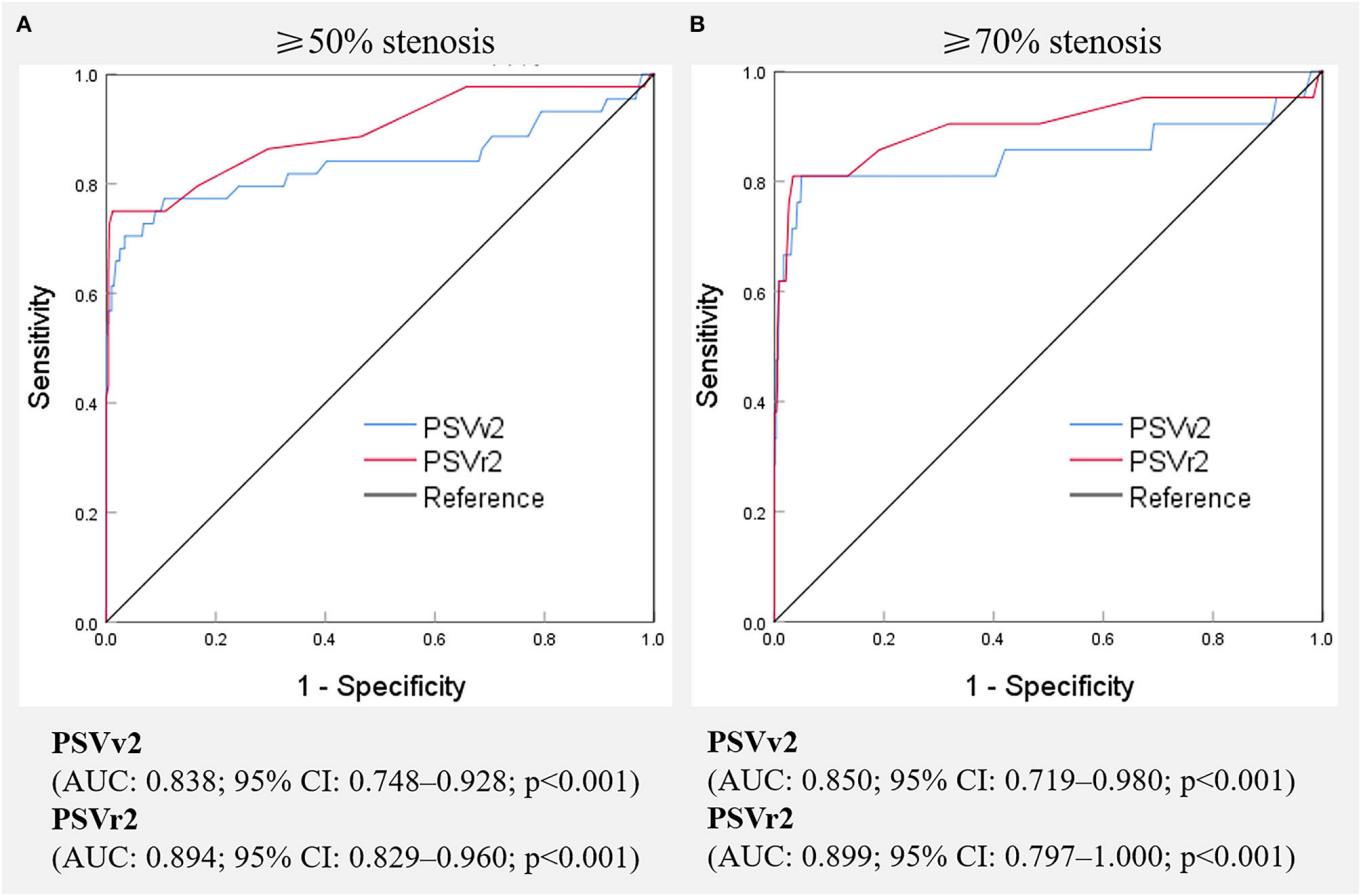
Receiver operating characteristic curves of various hemodynamic parameters for predicting ≥50% **(A)** and ≥70% **(B)** stenosis of the V2 segment of the vertebral artery. PSVv2, peak systolic velocity at V2 segment of vertebral artery; PSVr2, the ratio of peak systolic velocity at V2 segment to that at V1 segment of vertebral artery; AUC, the area under receiver operating characteristic curve, CI, confidence interval.

Digital subtraction angiography measurement of the narrowed lumen of the V2 segment of VA showed that 44 arteries (8.1%) had ≥50% stenosis, 21 arteries (3.9%) had ≥70% stenosis, and 500 arteries (91.9%) had normal lumen. DUS showed luminal narrowing, the increased velocity at the site of the lesion, and a relatively low-velocity flow in the V1 segment of VA. The ROC curves and AUCs of various hemodynamic parameters for evaluating ≥50% and ≥70% stenosis of the V2 segment of VA are shown in Figure 4; all AUCs were >0.80. For evaluating ≥50% stenosis, there was no significant difference between the AUCs of PSVv2 and PSVr2 (*P* = 0.0672). The cutoff values were PSVv2 ≥80 cm/s and PSVr2 ≥1.2. For evaluating ≥70% stenosis, there was no significant difference between the AUCs of PSVv2 and PSVr2 (*P* = 0.0942). The cutoff values were PSVv2 ≥111 cm/s and PSVr2 ≥1.7. The predictive values of cutoff values are shown in Table 3.

**Table 3:** Optimal cutoff values for the evaluation of the V2 segment of the vertebral artery.

	**Sensitivity (%)**	**Specificity (%)**	**PPV** **(%)**	**NPV (%)**	**Accuracy (%)**
≥50% stenosis
PSVv2 ≥ 80 cm/s	75.0	91.0	42.3	97.6	89.7
PSVr2 ≥ 1.2	75.0	94.8	55.9	97.7	93.2
≥70% stenosis
PSVv2 ≥ 111 cm/s	81.0	95.0	39.5	99.2	94.5
PSVr2 ≥ 1.7	81.0	96.6	48.6	99.2	96.0

*PPV, positive predictive value; NPV, negative predictive value; PSVv2, peak systolic velocity at V2 segment of vertebral artery; PSVr2, the ratio of peak systolic velocity at V2 segment to that of the V1 segment of the vertebral artery*.

## Discussion

In this study, we prospectively enrolled patients with symptomatic posterior circulation stroke/TIA who were scheduled to undergo DSA and aimed to validate the sensitivity and specificity of DUS and assess the optimal thresholds for ≥50% and ≥70% extracranial VA stenosis. In addition, the PPV, NPV, and accuracy were calculated to comprehensively validate the efficiency of DUS. The results showed that DUS was an effective non-invasive method for evaluating extracranial VA stenosis. Furthermore, we recommended a set of sonographic hemodynamic values to predict extracranial VA stenosis. The recommended graded cutoff values can help in the long-term management of patients with extracranial VA stenosis.

### Incidence of Extracranial VA Stenosis

In the current study, we examined 728 extracranial VAs in 364 patients and as reported in other studies, the V1 segment was the most common site of atherosclerotic stenosis in extracranial VA (27). There are few studies on V2 and V3 segment stenosis. In the Vertebral Artery Ischemia Stenting Trial (VIST), 179 patients presenting with posterior circulation TIA or non-disabling stroke and VA stenosis resulting from presumed atheromatous disease with stenosis ≥50% were included, and the stenosis of the V2/V3 segment of VA was found in 3.9% patients (11). In the current study, 728 extracranial VAs in patients with symptomatic posterior circulation stroke/TIA who were scheduled to undergo DSA were enrolled, and the incidence of V2 segment stenosis was 6.6%, and that of V3 segment stenosis was 0.1%. In addition, VA hypoplasia was frequent in this study, especially right VA hypoplasia, which is similar to a previous study (28). Park et al. (28) found VA hypoplasia was not rare in the normal population (26.5%) and was frequent in patients with posterior circulation strokes (45.6%). People with VA hypoplasia may have a high probability of posterior circulation strokes, with atherosclerotic susceptibility and ipsilateral lesions in the vertebral artery territory. Our study enrolled 364 patients with symptomatic posterior circulation stroke/TIA who were scheduled to undergo DSA and found VA hypoplasia was detected in about half of the enrolled patients.

### The Feasibility of Adequate Assessment of Extracranial VA Using DUS

Non-invasive assessment of VA stenosis is important because DSA is only necessary for patients with symptomatic VA disease who are potential candidates for revascularization. Appropriate noninvasive examination methods are needed to screen patients suitable for DSA. In patients with known VA stenosis, it is also reasonable to use a non-invasive examination to assess stenosis progression and to follow patients after revascularization therapies. V1 segment stenosis is often overestimated by magnetic resonance angiography, while computed tomography angiography underestimates the degree and prevalence of V1 segment stenosis (1). Previous studies indicated that duplex evaluation of extracranial VA was effective, but it was limited by vessel depth, tortuosity, and calcifications. Rozeman et al. (25) considered that the usefulness of DUS in screening for extracranial VA stenosis was limited since the adequate assessment of the V1 segment was often impossible due to anatomical difficulties and the low incidence of V2 segment stenosis. It should be pointed out that Rozeman et al. completed an adequate measurement of 56.1% extracranial VAs, possibly because they only used a compound imaging 9–3-MHz linear array transducer. In the current study, we examined 728 extracranial VAs in 364 Asian patients, and all V2 segments and 99.5% of V1 segments were visualized with the combined use of linear and curvilinear array probes. In addition, Rice et al. enrolled 243 VAs in 139 American patients and only 20 (8.2%) of extracranial VAs were not visualized by DSA or DUS with a curvilinear array probe (15). Yurdakul et al. enrolled 48 Turkish patients and only 2 (2.1%) extracranial VA could not be completely visualized by DUS with a 2.5- to 7-MHz linear and 1.5- to 4.5-MHz curvilinear probe (23). Therefore, with proper selection of probe, extracranial VA could be visualized adequately by DUS in patients from different continents, although they have different body mass indexes.

### Velocity Evaluation of Extracranial VA Stenosis

Currently, the velocity criteria for predicting stenosis of the V1 segment of VA are not uniform. There are several studies on ultrasonic evaluation of ≥50% stenosis of the V1 segment. Some studies considered PSVv1 to be more accurate, while others considered PSVr1; yet another study found that PSVv1 and PSVr1 appeared to be equally accurate. They recommended the PSVv1 cutoff value ranging from 90 cm/s to 140 cm/s and the PSVr1 cutoff value ranging from 2.1 to 2.2 (16, 22–25). In our study, we validated that PSVv1 was as accurate as PSVr1, and the cutoff values of PSVv1 ≥146 cm/s and PSVr1 ≥2.2 for detecting ≥50% stenosis, which was similar to previous studies. Moreover, to the best of our knowledge, there are only two studies that analyzed parameters useful for detecting ≥70% stenosis and had quite different results. Rice et al. enrolled 218 VAs and suggested that PSVv1 might have better sensitivity, and recommended the cutoff values of PSVv1 ≥110 cm/s (AUC: 0.84) (15). Hua et al. enrolled 247 VAs and suggested that PSVv1 was the most useful hemodynamic parameter and recommended using PSVv1 ≥210 cm/s (AUC: 0.950) and PSVr1 ≥4.0 (AUC: 0.907) as the cutoff values (16). In the current study, we conducted a prospective study with a large sample size to validate this controversy. We enrolled 544 VAs and found that PSVv1 was as accurate as PSVr1. The optimal cutoff values were PSVv1 ≥184 cm/s (AUC: 0.884) and PSVr1 ≥3.5 (AUC: 0.898). We consider that the differences may be partly related to different inclusion and exclusion criteria. Rice et al. reviewed patients who underwent DUS and DSA within 90 days, regardless of whether they had posterior circulation symptoms or coexistence of another stenosis (15). Hua et al. reviewed patients with symptoms of posterior circulation ischemia and excluded patients with anterior circulation stenosis, contralateral VA stenosis, tandem stenosis, and VA hypoplasia. In the current study, we prospectively enrolled patients with symptomatic posterior circulation stroke/TIA who were scheduled to undergo DSA and only excluded cases whose VA flow could not be measured. In this study, we attempted to seek patients with symptomatic VA disease who are potential candidates for revascularization in a real-world vascular study.

There is a paucity of published literature on ultrasonic evaluation of the V2 segment of VA. DUS examination cannot detect the V2 segment completely. Due to the acoustic shadow from the vertebral bone, the V2 segment can be insonated only in the intervertebral segment. However, the DUS examination still showed favorable diagnostic efficacy in V2 segment stenosis, with the AUCs of all parameters exceeding 0.8. Kuhl et al. found a mean PSVv2 of 49.9 ± 10.6 cm/s (range 30–80 cm/s) in the normal intertransverse V2 segment (20). Similarly, PSVv2 of 80 cm/s was also the cutoff value to predict ≥50% V2 segment stenosis in the current study. In addition, we recommended the cutoff values to predict ≥70% stenosis of the V2 segment, with DSA as the reference, which had not been reported previously, to the best of our knowledge.

### Clinical Significance of the Velocity Criteria

Symptomatic VA stenosis is a strong independent predictor of stroke recurrence (2). The high early risk of stroke provides a strong rationale for intensive therapy. Recent randomized controlled trials (SAMMPRIS, VISSIT, VAST, and VIST) showed that aggressive medical management was superior to stenting for intracranial VA stenosis, while endovascular treatment for extracranial VA stenosis appears safe and beneficial with low complications (7–10). In addition, V1 and V2 segments are amenable to endovascular therapy due to favorable anatomic features such as their short distance from the subclavian artery and their rarely tortuous nature. Endovascular treatment of the V3 segment is relatively risky due to the vessel tortuosity and redundancy that allow the mobility of the alanto-axial and alanto-occipital joint (12). Symptomatic patients with ≥50% extracranial VA stenosis despite optimal medical therapy and control of risk factors are managed with endovascular treatment. In addition, a few groups of asymptomatic patients with an elevated risk of stroke are also reasonable candidates for revascularization (13). Previous studies showed that stroke patients with ≥70% responsible artery stenosis are at the highest risk of stroke recurrence (3–6). The prime candidates for revascularization of extracranial VA stenosis are the patients with symptomatic vertebrobasilar insufficiency and bilateral ≥70% VA stenosis, unilateral ≥70% VA stenosis in the presence of an occluded or hypoplastic contralateral VA, artery-to-artery embolism even in the presence of unilateral VA stenosis, or significant dependence on collateral flow from the posterior circulation, such as in cases of carotid occlusion (12). Therefore, noninvasive evaluation of V1 and V2 segments stenosis is important, especially ≥70% stenosis.

In the current study, the cutoff values for predicting ≥50% and ≥70% V1 segment stenosis were PSVv1 ≥146 cm/s and PSVr1 ≥2.2, and PSVv1 ≥184 cm/s and PSVr1 ≥3.5, respectively. The cutoff values for predicting ≥50% and ≥70% V2 segment stenosis were PSVv2 ≥80 cm/s and PSVr2 ≥1.2, and PSVv2 ≥111 cm/s and PSVr2 ≥1.7, respectively. Therefore, if the patient has symptoms of vertebrobasilar insufficiency, and the ultrasonic parameters of PSVv1 ≥146 cm/s and PSVr1 ≥2.2 at the V1 segment, or PSVv2 ≥80 cm/s and PSVr2 ≥1.2 at the V2 segment, they should be considered for further verification by DSA to seek revascularization. If the parameters increase to PSVv1 ≥184 cm/s and PSVr1 ≥3.5 at the V1 segment, or PSVv2 ≥111 cm/s and PSVr2 ≥1.7 at the V2 segment, the patient has an increased risk of recurrent stroke and is more likely to need revascularization. In addition, the cutoff values for ≥70% stenosis achieved better specificity and NPV than cutoff values for ≥50% stenosis in the current study. DUS is often a first step in the vascular workup for the evaluation of vascular stenosis. It is appropriate as a screening tool for symptomatic VA diseases in the clinical setting (1). As a screening tool, the parameters with high specificity and NPV would be ideal. Therefore, DUS may be more suitable for the screening of ≥70% VA stenosis.

### Limitation

Our study has limitations. First, this was a prospective, single-center study, which may cause a variety of selection biases that could affect disease prevalence. However, the single-center design is advantageous in terms of sample homogeneity. All DUS were performed by the same physician, and all angiographies were interpreted by the same neuroendovascular physicians. Nevertheless, we aim to conduct multicentre studies to further verify the efficacy of our results in the future. Second, to validate the conflicting threshold of predicting V1 segment ≥70% stenosis accurately, we enrolled patients with symptomatic posterior circulation stroke/TIA who had undergone CTA or MRA which confirmed VA stenosis, and who were scheduled to undergo subsequent DSA as part of interventional therapy. By selecting only candidates for DSA in our study population, we would overestimate the prevalence of higher-grade extracranial VA stenosis. This may affect the accurate estimations of PPV and NPV.

In conclusion, our study found that DUS is a reliable imaging modality for evaluating extracranial VA stenosis. Symptomatic patients with the ultrasonic parameters of PSVv1 ≥146 cm/s and PSVr1 ≥2.2 at the V1 segment or PSVv2 ≥80 cm/s and PSVr2 ≥1.2 at the V2 segment need to be considered for further verification by DSA to seek revascularization. If the parameters increase to PSVv1 ≥184 cm/s and PSVr1 ≥3.5 at the V1 segment or PSVv2 ≥111 cm/s and PSVr2 ≥1.7 at the V2 segment, these patients have an increased risk of recurrent stroke and are more likely to need revascularization. The results can be used as a reference for the assessment and long-term management of patients with extracranial VA stenosis.

## Data Availability Statement

The raw data supporting the conclusions of this article will be made available by the authors, without undue reservation.

## Ethics Statement

The studies involving human participants were reviewed and approved by Ethics Committee of the First Hospital of Jilin University. The patients/participants provided their written informed consent to participate in this study.

## Author Contributions

JZ contributed to the study conception and design, data collection, analysis and interpretation, and drafting of the manuscript. YX and LC contributed to the study conception and design, analysis and interpretation of the data, and revision of the manuscript. All the authors contributed to the article and approved the submitted version.

## Conflict of Interest

The authors declare that the research was conducted in the absence of any commercial or financial relationships that could be construed as a potential conflict of interest.

## Publisher's Note

All claims expressed in this article are solely those of the authors and do not necessarily represent those of their affiliated organizations, or those of the publisher, the editors and the reviewers. Any product that may be evaluated in this article, or claim that may be made by its manufacturer, is not guaranteed or endorsed by the publisher.
